# Changes in sexual attitudes and lifestyles in Britain through the life course and over time: findings from the National Surveys of Sexual Attitudes and Lifestyles (Natsal)

**DOI:** 10.1016/S0140-6736(13)62035-8

**Published:** 2013-11-30

**Authors:** Catherine H Mercer, Clare Tanton, Philip Prah, Bob Erens, Pam Sonnenberg, Soazig Clifton, Wendy Macdowall, Ruth Lewis, Nigel Field, Jessica Datta, Andrew J Copas, Andrew Phelps, Kaye Wellings, Anne M Johnson

**Affiliations:** a Research Department of Infection and Population Health, University College London, London, UK; b Department of Health Services Research and Policy, London School of Hygiene and Tropical Medicine, London, UK; c Department of Social and Environmental Health Research, London School of Hygiene and Tropical Medicine, London, UK; d NatCen Social Research, London, UK

## Abstract

**Background:**

Sexual behaviour and relationships are key components of wellbeing and are affected by social norms, attitudes, and health. We present data on sexual behaviours and attitudes in Britain (England, Scotland, and Wales) from the three National Surveys of Sexual Attitudes and Lifestyles (Natsal).

**Methods:**

We used a multistage, clustered, and stratified probability sample design. Within each of the 1727 sampled postcode sectors for Natsal-3, 30 or 36 addresses were randomly selected and then assigned to interviewers. To oversample individuals aged 16–34 years, we randomly allocated addresses to either the core sample (in which individuals aged 16–74 years were eligible) or the boost sample (in which only individuals aged 16–34 years were eligible). Interviewers visited all sampled addresses between Sept 6, 2010, and Aug 31, 2012, and randomly selected one eligible individual from each household to be invited to participate. Participants completed the survey in their own homes through computer-assisted face-to-face interviews and self-interview. We analysed data from this survey, weighted to account for unequal selection probabilities and non-response to correct for differences in sex, age group, and region according to 2011 Census figures. We then compared data from participants aged 16–44 years from Natsal-1 (1990–91), Natsal-2 (1999–2001), and Natsal-3.

**Findings:**

Interviews were completed with 15 162 participants (6293 men, 8869 women) from 26 274 eligible addresses (57·7%). 82·1% (95% CI 81·0–83·1%) of men and 77·7% (76·7–78·7%) of women reported at least one sexual partner of the opposite sex in the past year. The proportion generally decreased with age, as did the range of sexual practices with partners of the opposite sex, especially in women. The increased sexual activity and diversity reported in Natsal-2 in individuals aged 16–44 years when compared with Natsal-1 has generally been sustained in Natsal-3, but in men has generally not risen further. However, in women, the number of male sexual partners over the lifetime (age-adjusted odds ratio 1·18, 95% CI 1·08–1·28), proportion reporting ever having had a sexual experience with genital contact with another woman (1·69, 1·43–2·00), and proportion reporting at least one female sexual partner in the past 5 years (2·00, 1·59–2·51) increased in Natsal-3 compared with Natsal-2. While reported number of occasions of heterosexual intercourse in the past 4 weeks had reduced since Natsal-2, we recorded an expansion of heterosexual repertoires—particularly in oral and anal sex—over time. Acceptance of same-sex partnerships and intolerance of non-exclusivity in marriage increased in men and women in Natsal-3.

**Interpretation:**

Sexual lifestyles in Britain have changed substantially in the past 60 years, with changes in behaviour seeming greater in women than men. The continuation of sexual activity into later life—albeit reduced in range and frequency—emphasises that attention to sexual health and wellbeing is needed throughout the life course.

**Funding:**

Grants from the UK Medical Research Council and the Wellcome Trust, with support from the Economic and Social Research Council and the Department of Health.

## Introduction

Improving sexual and reproductive health remains a public health priority in Britain (England, Scotland, and Wales),^1^ as it does globally.^2^ A range of factors contribute to a population's sexual health, such as social context and the interplay between behaviour, relationships, and health status.^3^ People younger than 25 years are at highest risk for some adverse sexual health outcomes, such as sexually transmitted infections^4^ and unplanned pregnancies.^5^ However, research into the sexual health and wellbeing of men and women in later life—who now have increasing expectations of sexual fulfilment^6^ and make up a growing segment of the population—is a neglected area.^7^, ^8^

The first National Survey of Sexual Attitudes and Lifestyles (Natsal-1) was done in a probability sample of 18 876 adults aged 16–59 years in Britain in 1990–91.^9^, ^10^ It provided urgently needed population-based data to inform the prevention and prediction of HIV transmission.^9^, ^10^ A second survey (Natsal-2) of 11 161 adults aged 16–44 years in 1999–2001 extended the investigative focus to broader aspects of sexual and reproductive health.^11^, ^12^, ^13^, ^14^ Data from these surveys have been widely used to inform sexual and reproductive health policy in Britain.^15^, ^16^, ^17^, ^18^, ^19^

Here, we report data on sexual behaviours and attitudes in Britain from the latest survey, Natsal-3, and the two previous surveys. The combination of data from all three Natsal surveys enables both period and birth cohort analyses; together, the surveys sampled people born between the 1930s and the 1990s. We examine changes in sexual lifestyles throughout the life course and trends over time.

## Methods

### Participants and procedures

Full details of the methods used in Natsal-3 have been reported elsewhere.^20^, ^21^ Briefly, we used a multistage, clustered, and stratified probability sample design. 1727 postcode sectors (geographical units used for sorting mail) throughout Britain were used as the primary sampling units and were randomly allocated to one of eight periods of fieldwork that took place between Sept 6, 2010, and Aug 31, 2012, with each period lasting about 3 months.

Within each primary sampling unit, 30 or 36 addresses were randomly selected and then assigned to interviewers from NatCen Social Research. To allow detailed exploration of behaviours in the age group at highest risk of some sexual health outcomes (eg, unplanned pregnancy and sexually transmitted infections), we oversampled individuals aged 16–34 years. We randomly allocated addresses to either the core sample (in which all individuals aged 16–74 years were eligible) or the boost sample (in which only individuals aged 16–34 years were eligible). Letters and leaflets giving background information about Natsal-3 were sent to sampled addresses before visits began.

Interviewers visited all sampled addresses, identified residents in the eligible age range, and randomly selected one individual to be invited to participate in the survey using a Kish grid technique. Participants then completed the survey in their own homes through a combination of face-to-face interviews with computer-assisted personal interview and a self-completion format with computer-assisted self-interview. Interviewers were present in the room while participants completed the computer-assisted self-interview and could provide assistance as necessary, but did not view responses. On completion of computer-assisted self-interviews, answers could not be accessed by interviewers. No names or other potentially identifying information was attached to the interviews. Interviews lasted about 1 h on average. Participants received a £15 gift voucher as a token of appreciation. An anonymised dataset will be deposited with the UK Data Archive, and the complete questionnaire and technical report will be available on the Natsal website on the day of publication.

These methods are broadly the same as those used in Natsal-1 and Natsal-2. However, in Natsal-1, paper was used (rather than computers) during face-to-face interviews and self-interview. Descriptions of the development stages of previous surveys are available elsewhere.^9^, ^10^, ^11^, ^22^, ^23^, ^24^ Most questions in Natsal-3 were identical to those in Natsal-1 and Natsal-2, including questions about age and circumstances of first sexual intercourse, sexual practices, experiences with sexual partners, details of most recent partners, and attitudes. Questions added in Natsal-3 included those about health problems that could affect sexual lifestyles, sexual function and satisfaction, a screen for depressive symptoms, non-volitional sex, and unplanned pregnancy.^20^, ^21^ The questionnaire underwent thorough cognitive testing and piloting, as previously reported.^25^

As in Natsal-1 and Natsal-2, we weighted Natsal-3 data to adjust for the unequal probabilities of selection in terms of age and the number of adults in the eligible age range at an address. After application of these selection weights, the Natsal-3 sample was broadly representative of the British population compared with 2011 Census figures,^26^, ^27^ although men and London residents were slightly under-represented. Therefore, as in previous surveys, we also applied a non-response post-stratification weight to correct for differences in sex, age, and Government Office Region between the achieved sample and the 2011 Census (appendix).^26^, ^27^

We compared data for participants aged 16–44 years in each survey. This age group was common to all three surveys. Information about variables that were compared was derived from identically worded questions. All three surveys had been weighted for differential selection probabilities. Natsal-1 was post-stratified to 1991 Census figures and Natsal-2 to 2001 Census figures, with procedures described for Natsal-3,^20^, ^21^ which allowed us to make comparisons between the three surveys. However, there are minor differences from the weighting schemes used in previous reports.^9^, ^10^, ^11^, ^12^, ^13^, ^23^

The Natsal-3 study was approved by the Oxfordshire Research Ethics Committee A (reference: 09/H0604/27). Participants provided oral informed consent for interviews.

### Statistical analysis

We did all analyses with the complex survey functions of Stata (version 12.1) to incorporate weighting, clustering, and stratification of data. We present descriptive statistics by sex and age group. We used binary logistic regression to calculate age-adjusted odds ratios (aAORs) to investigate how reporting of key sexual behaviours and attitudes varied by three measures of social status: the National Statistics Socio-Economic Classification^28^ (NS-SEC; individual level), education attainment defined according to school leaving age and academic qualifications obtained (individual level), and the Index of Multiple Deprivation^29^ (area level). We then used ordinal and binary logistic regression to calculate aAORs to compare estimates between the three Natsal surveys for participants aged 16–44 years. We used Natsal-2 as the reference category to quantify the change between Natsal-2 and Natsal-3 and also to allow comparisons with Natsal-1. Finally, we obtained data from the three surveys to examine changes in the reporting of sexual practices with partners of the opposite sex and partner numbers by birth cohort and age at interview. We used an α of 0·05 in all analyses.

### Role of the funding source

The sponsors of the study had no role in study design, data collection, data analysis, data interpretation, or writing of the report. The corresponding author had full access to all the data in the study and had final responsibility for the decision to submit for publication.

## Results

A resident within the eligible age range (16–74 years for the core sample or 16–34 years for the boost sample) was identified at 23 360 of 59 412 addresses selected for sampling. Eligibility could not be established at 4143 addresses (7·0%), because contact was attempted on several occasions but could not be made or all information was refused. By assuming the percentage of residents who would be eligible at the addresses for which this percentage was unknown was the same as for the addresses for which eligibility was known (separately for core and boost addresses), we estimated that 2914 of these addresses would have housed a resident in the eligible age range, giving an estimated total of 26 274 eligible addresses.

The response rate was 57·7%; interviews were completed with 15 162 participants (6293 men, 8869 women) from the 26 274 eligible addresses. Individuals from 6343 eligible addresses refused to participate, contact was not made at 327 addresses, and interviews were not completed for 1528 for other reasons. Therefore, the co-operation rate (how many interviews were completed from eligible addresses for which contact was made) was 65·8%.^20^, ^21^, ^30^ The demographic characteristics of participants in the Natsal-3 survey after final weighting are shown in table 1. Notably, the proportion of participants aged 16–44 years living with a partner (married or unmarried couples) has decreased to 52·0% of men and 54·5% of women in Natsal-3 from 57·7% of men and 64·7% of women of this age in Natsal-1.^11^

**Table 1:** Demographic characteristics of participants in Natsal-3, by sex and age group

	**Men**							**Women**						
	16–24 years	25–34 years	35–44 years	45–54 years	55–64 years	65–74 years	All age groups	16–24 years	25–34 years	35–44 years	45–54 years	55–64 years	65–74 years	All age groups
**Marital status**														
Married or civil partnership	1·7%	34·6%	66·1%	64·5%	67·9%	73·8%	50·6%	2·9%	41·4%	62·5%	62·0%	66·4%	60·1%	49·7%
Cohabitation (with partner of opposite or same sex)	9·4%	26·1%	13·4%	11·6%	6·8%	3·5%	12·5%	11·2%	22·7%	13·9%	7·5%	5·2%	2·8%	11·4%
Previously married or had civil partner	0·1%	2·1%	6·4%	12·5%	17·0%	18·3%	8·8%	0·2%	4·3%	11·4%	21·3%	23·0%	34·2%	14·9%
Single and never married	88·9%	37·2%	14·1%	11·4%	8·3%	4·4%	28·2%	85·7%	31·7%	12·2%	9·3%	5·4%	2·8%	24·0%
**Ethnic origin**														
White	84·4%	79·9%	84·7%	89·9%	94·2%	95·7%	87·5%	82·3%	82·7%	86·5%	88·7%	92·6%	96·4%	87·8%
Mixed	3·1%	3·1%	0·9%	0·9%	0·7%	0·6%	1·6%	4·0%	2·2%	1·9%	1·4%	0·8%	0·5%	1·8%
Asian or Asian British	8·4%	11·4%	9·4%	4·7%	3·3%	2·4%	7·0%	7·8%	10·1%	7·0%	4·0%	4·4%	1·8%	6·1%
Black or black British	3·4%	4·3%	4·1%	3·5%	1·5%	1·2%	3·2%	4·7%	4·2%	3·9%	5·3%	1·7%	1·3%	3·7%
Other	0·8%	1·4%	0·8%	1·0%	0·3%	0·0%	0·8%	1·2%	0·8%	0·7%	0·6%	0·6%	0·1%	0·7%
**Self-defined sexual identity**														
Heterosexual/straight	96·7%	96·5%	97·7%	96·7%	96·8%	99·0%	97·1%	95·9%	96·6%	96·8%	97·4%	99·1%	99·4%	97·3%
Gay/lesbian	1·5%	2·4%	1·3%	1·8%	1·3%	0·2%	1·5%	1·2%	1·2%	1·5%	1·1%	0·8%	0·1%	1·0%
Bisexual	1·5%	0·7%	0·9%	1·2%	1·3%	0·5%	1·0%	2·5%	2·0%	1·4%	1·0%	0%	0·3%	1·4%
Other	0·3%	0·5%	0·1%	0·3%	0·6%	0·3%	0·3%	0·5%	0·2%	0·3%	0·5%	0·1%	0·3%	0·3%
Academic qualifications*														
No academic qualifications	6·6%	9·0%	14·1%	17·4%	37·1%	52·3%	21·0%	6·1%	9·1%	9·9%	17·2%	38·8%	54·6%	21·0%
Academic qualifications typically gained at age 16 years†	26·7%	32·9%	42·2%	40·5%	25·2%	18·7%	32·3%	25·4%	31·1%	42·5%	41·0%	33·7%	27·5%	34·4%
Studying for or have attained further academic qualifications	66·7%	58·1%	43·7%	42·1%	37·7%	29·0%	46·7%	68·5%	59·9%	47·6%	41·7%	27·5%	18·0%	44·7%
**National Statistics Socio-Economic Classification**^28^														
Managerial and professional occupations	9·2%	42·2%	42·5%	44·2%	40·9%	26·2%	35·1%	9·6%	37·7%	41·6%	39·0%	31·0%	14·9%	30·4%
Intermediate occupations	7·8%	16·8%	18·4%	19·9%	19·4%	17·3%	16·7%	9·0%	22·6%	21·6%	23·1%	21·9%	14·8%	19·5%
Semiroutine and routine occupations	31·1%	34·1%	35·9%	30·5%	30·7%	28·7%	32·1%	29·1%	28·0%	25·7%	25·5%	29·1%	20·3%	26·2%
Never worked and long-term unemployed	6·3%	1·4%	2·2%	4·3%	8·9%	27·4%	7·1%	7·3%	6·9%	9·3%	11·5%	17·7%	49·9%	15·4%
Full-time students	45·6%	5·5%	0·9%	1·1%	0·0%	0·4%	9·0%	45·1%	4·8%	1·8%	0·9%	0·3%	0·2%	8·5%
**Quintile of Index of Multiple Deprivation**^29^														
1 (least deprived)	17·5%	14·5%	20·9%	22·8%	22·2%	25·7%	20·3%	17·0%	13·3%	20·8%	23·1%	22·5%	24·7%	20·0%
2	19·1%	16·7%	21·1%	21·9%	24·6%	25·9%	21·2%	18·3%	16·7%	21·1%	20·6%	23·1%	24·5%	20·4%
3	18·4%	19·8%	19·9%	18·5%	21·1%	18·7%	19·4%	19·4%	20·6%	19·8%	19·5%	20·1%	17·8%	19·8%
4	22·9%	23·6%	21·4%	17·7%	15·6%	17·4%	20·0%	23·0%	24·1%	20·1%	17·3%	19·9%	16·4%	20·3%
5 (most deprived)	22·2%	25·3%	16·6%	19·2%	16·5%	12·3%	19·1%	22·4%	25·2%	18·2%	19·5%	14·3%	16·6%	19·6%
**Denominators**														
Unweighted	1729	1525	806	794	772	667	6293	2140	2487	1215	1123	1030	874	8869
Weighted	1238	1374	1425	1413	1197	860	7508	1207	1380	1455	1443	1235	935	7654

All participants (denominators vary across variables because of item non-response).

* Participants aged ≥17 years.

† English General Certificate of Secondary Education or equivalent.

Median age at first heterosexual intercourse was 17 years in both sexes (Table 2, Table 3), but was 16 years (IQR 15–18) in those aged 16–24 years at interview. The proportion reporting first heterosexual intercourse before age 16 years increased in successive birth cohorts in both men and women (Table 2, Table 3). The difference between the sexes in age at first heterosexual intercourse in the older age groups was not recorded in individuals aged 16–34 years (Table 2, Table 3).

**Table 2:** Sexual partners, practices, behaviours, and attitudes reported by men in Natsal-3, by age group

		**16–24 years**	**25–34 years**	**35–44 years**	**45–54 years**	**55–64 years**	**65–74 years**	**All age groups**	**p value**
Age at first heterosexual intercourse*									
Age (years)		16 (15–18)	17 (15–19)	17 (15–19)	17 (15–18)	18 (16–19)	18 (16–21)	17 (16–19)	..
Heterosexual intercourse before age 16 years		30·9% (28·5–33·5%)	25·5% (23·1–28·1%)	26·6% (23·4–30·1%)	26·7% (23·4–30·3%)	17·3% (14·6–20·5%)	15·4% (12·6–18·6%)	24·4% (23·1–25·7%)	<0·0001
**Female sexual partners**									
Number of partners over the lifetime									<0·0001
	0	19·8% (17·8–22·0%)	5·2% (4·0–6·7%)	1·5% (0·8–2·9%)	2·0% (1·2–3·3%)	2·2% (1·4–3·4%)	1·8% (0·9–3·4%)	5·5% (5·0–6·1%)	..
	1	15·4% (13·7–17·2%)	12·6% (10·7–14·8%)	11·8% (9·4–14·6%)	8·8% (6·7–11·4%)	15·3% (12·4–18·8)	22·9% (19·5–26·7%)	13·7% (12·7–14·8%)	..
	2	11·8% (10·0–13·8%)	6·7% (5·4–8·3%)	5·7% (4·0–8·0%)	7·2% (5·3–9·7%)	9·3% (7·2–12·0%)	11·2% (8·6–14·5%)	8·4% (7·5–9·3%)	..
	3–4	15·7% (13·8–18·0%)	14·3% (12·4–16·4%)	12·4% (10·0–15·4)	13·6% (11·1–16·4%)	17·6% (14·6–21·1%)	18·4% (15·2–22·0%)	15·0% (13·9–16·1%)	..
	5–9	17·6% (15·7–19·8%)	23·5% (21·2–26·0%)	26·5% (23·2–30·0%)	25·4% (22·1–29·0%)	23·9% (20·7–27·5%)	23·4% (19·8–27·4%)	23·5% (22·3–24·8%)	..
	≥10	19·6% (17·6–21·8%)	37·8% (35·1–40·6%)	42·1% (38·5–45·9%)	43·1% (39·4–46·9%)	31·7% (28·1–35·5%)	22·3% (19·0–26·0%)	33·9% (32·6–35·4%)	..
	Mean (SD)	6·5 (13·3)	13·7 (23·9)	14·3 (24·2)	17·5 (70·4)	20·1 (149·2)	12·1 (45)	14·1 (69·6)	..
	Median (IQR)	3 (1–7)	6 (3–15)	8 (4–18)	8 (4–20)	5 (2–11)	4 (2–8)	6 (2–12)	..
Number of partners in past year									<0·0001
	0	24·1% (21·9–26·5%)	9·9% (8·3–11·9%)	7·5% (5·8–9·6%)	13·6% (11·3–16·2%)	23·7% (20·7–27·0%)	40·2% (36·2–44·3%)	17·9% (16·9–19·0%)	..
	1	42·2% (39·5–45·0%)	71·0% (68·2–73·5%)	81·8% (78·8–84·4%)	76·0% (72·8–79·0%)	68·3% (64·6–71·7%)	56·3% (52·1–60·4%)	67·1% (65·8–68·5%)	..
	≥2	33·7% (31·2–36·3%)	19·1% (17·0–21·3%)	10·7% (8·7–13·2%)	10·4% (8·4–12·9%)	8·1% (6·2–10·5%)	3·5% (2·3–5·4%)	14·9% (14·0–15·9%)	..
At least one new partner in past year		46·0% (43·1–48·8%)	26·3% (24·0–28·8%)	13·2% (11·0–15·8%)	12·3% (10·1–14·9%)	10·4% (8·4–12·9%)	5·0% (3·5–7·3%)	19·7% (18·7–20·8%)	<0·0001
**Sexual practices with female partners**									
Number of occasions of sexual intercourse in past 4 weeks†									
	Mean (SD)	5·1 (7·2)	5·4 (6·5)	4·1 (4·3)	4·1 (6·1)	3·2 (4·5)	2·3 (3·6)	4·3 (5·7)	..
	Median (IQR)	3 (0–7)	4 (1–8)	3 (1–6)	3 (1–6)	2 (0–4)	1 (0–3)	3 (1–6)	..
Vaginal sex in past 4 weeks		52·7% (50·0–55·3%)	74·6% (72·1–76·9%)	75·1% (71·8–78·3%)	69·3% (65·7–72·7%)	55·2% (51·3–59·1%)	37·2% (33·1–41·5%)	62·9% (61·5–64·4%)	<0·0001
Given or received oral sex in past year		70·9% (68·4–73·3%)	80·0% (77·6–82·2%)	80·2% (77·0–83·0%)	71·0% (67·2–74·5%)	52·4% (48·4–56·4%)	30·4% (26·5–34·6%)	67·1% (65·7–68·5%)	<0·0001
Anal sex in past year		18·5% (16·4–20·7%)	17·6% (15·6–19·8%)	15·1% (12·6–18·0%)	13·8% (11·1–17·0%)	7·6% (5·7–10·0%)	2·9% (1·7–4·9%)	13·4% (12·4–14·4%)	<0·0001
Genital contact without intercourse in past year		71·3% (68·6–73·8%)	75·8% (73·1–78·3%)	73·1% (69·6–76·3%)	66·0% (62·0–69·8%)	56·2% (52·3–60·1%)	36·8% (32·7–41·1%)	65·4% (63·9–66·8%)	<0·0001
**Masturbation**									
Masturbated in past 4 weeks		82·6% (80·5–84·5%)	78·1% (75·6–80·3%)	72·9% (69·1–76·3%)	64·6% (60·6–68·4%)	53·1% (49·0–57·1%)	33·1% (29·2–37·3%)	66·4% (64·9–67·8%)	<0·0001
**Sexual practices with male partners**									
Any sexual experience or contact with another man		7·0% (5·7–8·5%)	7·8% (6·4–9·4%)	7·4% (5·5–9·8%)	9·2% (7·3–11·6%)	10·0% (8·0–12·4%)	6·1% (4·4–8·4%)	8·0% (7·2–8·9%)	0·0754
Any sexual experience with genital contact with another man		4·0% (3·1–5·2%)	5·7% (4·6–7·1%)	4·7% (3·3–6·6%)	7·2% (5·5–9·3%)	7·3% (5·6–9·5%)	3·4% (2·3–5·1%)	5·5% (4·9–6·2%)	0·0016
At least one male sexual partner in past 5 years		2·9% (2·1–3·9%)	3·5% (2·6–4·7%)	2·3% (1·4–3·7%)	2·9% (1·9–4·3%)	2·3% (1·3–3·8%)	0·9% (0·4–2·0%)	2·6% (2·1–3·0%)	0·0598
**Risk behaviours for HIV and sexually transmitted infections**‡									
Paid for sex§ in past 5 years		2·7% (1·9–3·7%)	5·4% (4·3–6·8%)	3·9% (2·7–5·7%)	3·6% (2·5–5·1%)	3·6% (2·4–5·4%)	1·2% (0·6–2·4%)	3·6% (3·1–4·2%)	0·0017
At least one new sexual partner from outside the UK in past 5 years		13·2% (11·5–15·2%)	14·5% (12·8–16·5%)	7·5% (5·6–9·9%)	4·5% (3·1–6·4%)	3·7% (2·5–5·5%)	1·3% (0·6–2·6%)	7·9% (7·2–8·8%)	<0·0001
At least two sexual partners with whom no condom used in past year		16·4% (14·5–18·4%)	10·1% (8·6–11·9%)	6·4% (4·9–8·5%)	5·8% (4·3–7·8%)	3·5% (2·4–5·1%)	0·6% (0·2–1·7%)	7·6% (6·9–8·3%)	<0·0001
**Sexual attitudes**									
Non-exclusivity in marriage: always wrong		66·5% (63·8–69·1%)	62·3% (59·4–65·1%)	59·2% (55·4–62·8%)	52·7% (48·7–56·7%)	47·7% (43·7–51·8%)	52·7% (48·4–56·9%)	57·2% (55·7–58·7%)	<0·0001
One night stands: not wrong at all		19·2% (17·0–21·5%)	21·7% (19·4–24·1%)	19·6% (16·6–22·9%)	16·8% (14·0–20·1%)	14·1% (11·7–16·8%)	10·2% (8·0–13·1%)	17·5% (16·3–18·7%)	<0·0001
Male same-sex partnerships: not wrong at all		48·6% (45·7–51·4%)	50·0% (47·0–52·9%)	46·0% (42·2–49·8%)	38·5% (34·8–42·4%)	33·3% (29·6–37·2%)	21·1% (17·9–24·8%)	41·0% (39·5–42·4%)	<0·0001
Female same-sex partnerships: not wrong at all		53·1% (50·3–56·0%)	54·3% (51·3–57·3%)	49·8% (46·0–53·7%)	42·9% (39·1–46·8%)	37·9% (34·1–41·9%)	24·4% (20·9–28·2%)	45·2% (43·8–46·7%)	<0·0001
**Denominator**									
Unweighted		1729	1525	806	794	772	667	6293	..
Weighted		1238	1374	1425	1413	1197	860	7508	..

Data are median (IQR), % (95% CI), or mean (SD), unless otherwise stated. All estimates are weighted. All participants (denominators vary across variables because of item non-response). Vaginal sex is defined as a man's penis in a woman's vagina.^21^ Oral sex is defined as mouth on a partner's genital area.^21^ Anal sex is defined as a man's penis in a partner's anus.^21^

* Not defined when asked in interview.

† Sexual intercourse defined as vaginal, oral, or anal sex; in participants who had at least one female sexual partner in the past year.

‡ With a female or male sexual partner, or both.

§ Vaginal, oral, or anal sex.

**Table 3:** Sexual partners, practices, behaviours, and attitudes reported by women in Natsal-3, by age group

		**16–24 years**	**25–34 years**	**35–44 years**	**45–54 years**	**55–64 years**	**65–74 years**	**All age groups**	**p value**
Age at first heterosexual intercourse*									
Age (years)		16 (15–18)	17 (15–18)	17 (16–19)	17 (16–19)	18 (17–20)	19 (17–21)	17 (15–22)	..
Heterosexual intercourse before age 16 years		29·2% (27·0–31·4%)	25·1% (23·3–27·1%)	18·1% (15·8–20·6%)	14·3% (12·2–16·7%)	9·9% (8·1–12·1%)	4·0% (2·8–5·9%)	17·4% (16·5–18·3%)	<0·0001
**Male sexual partners**									
Number of partners over the lifetime									<0·0001
	0	19·8% (17·8–21·8%)	2·6% (1·9–3·5%)	0·5% (0·2–1·5%)	0·6% (0·4–1·2%)	0·9% (0·6–1·6%)	0·9% (0·5–1·7%)	4·2% (3·8–4·6%)	..
	1	16·4% (14·7–18·4%)	18·1% (16·4–20·0%)	15·8% (13·6–18·3%)	19·7% (17·1–22·6%)	28·9% (25·9–32·2%)	43·4% (39·5–47·4%)	22·4% (21·3–23·5%)	..
	2	10·2% (8·9–11·7%)	8·2% (7·1–9·6%)	8·1% (6·5–10·0%)	10·8% (9·0–13·1%)	14·6% (12·3–17·2%)	17·6% (14·8–20·7%)	11·1% (10·3–11·9%)	..
	3–4	16·4% (14·6–18·3%)	16·4% (14·9–18·0%)	19·4% (17·1–21·9%)	22·0% (19·3–24·9%)	21·8% (19·1–24·7%)	19·1% (16·3–22·2%)	19·2% (18·2–20·2%)	..
	5–9	20·9% (18·9–22·9%)	24·9% (23·1–26·9%)	28·1% (25·5–30·9%)	27·9% (25·0–30·9%)	21·4% (18·8–24·1%)	10·9% (8·9–13·4%)	23·2% (22·2–24·3%)	..
	≥10	16·4% (14·6–18·2%)	29·8% (27·7–31·9%)	28·1% (25·4–30·9%)	18·9% (16·5–21·6%)	12·4% (10·4–14·8%)	8·0% (6·3–10·2%)	19·9% (19·0–20·9%)	..
	Mean (SD)	5·2 (8·1)	8·9 (17·2)	8·5 (19·7)	6·8 (11·8)	6·1 (38·3)	6·3 (73·9)	7·1 (32·1)	..
	Median (IQR)	3 (1–7)	5 (2–10)	5 (3–10)	4 (2–7)	3 (1–5)	2 (1–4)	4 (1–8)	..
Number of partners in past year									<0·0001
	0	23·0% (21·0–25·1%)	8·2% (7·0–9·5%)	9·2% (7·5–11·2%)	15·0% (13·1–17·2%)	36·3% (33·0–39·6%)	57·9% (54·0–61·7%)	22·3% (21·3–23·3%)	..
	1	50·3% (47·9–52·7%)	79·1% (77·1–80·9%)	82·9% (80·5–85·1%)	80·5% (78·1–82·6%)	62·3% (58·9–65·5%)	41·4% (37·7–45·3%)	68·4% (67·2–69·5%)	..
	≥2	26·7% (24·5–29·0%)	12·8% (11·3–14·3%)	7·9% (6·5–9·6%)	4·5% (3·5–5·8%)	1·5% (0·9–2·3%)	0·7% (0·3–1·7%)	9·3% (8·7–10·0%)	..
At least one new partner in past year		38·3% (35·9–40·7%)	19·6% (17·9–21·5%)	11·2% (9·5–13·2%)	8·9% (7·4–10·8%)	4·4% (3·3–5·9%)	2·1% (1·2–3·6%)	14·5% (13·8–15·4%)	<0·0001
**Sexual practices with male partners**									
Number of occasions of sexual intercourse in past 4 weeks†									
	Mean (SD)	5·8 (6·6)	4·9 (5·1)	4 (4·6)	3·5 (4·2)	2·5 (3·4)	1·4 (2·3)	4·0 (4·9)	..
	Median (IQR)	4 (1–8)	4 (1–7)	3 (1–5)	2 (1–5)	2 (0–4)	1 (0–2)	3 (1–6)	..
Vaginal sex in past 4 weeks		59·6% (57·2–61·9%)	75·7% (73·8–77·5%)	72·3% (69·4–74·9%)	64·4% (61·2–67·3%)	41·4% (38·1–44·7%)	21·3% (18·2–24·8%)	58·4% (57·2–59·7%)	<0·0001
Given or received oral sex in past year		70·3% (68·0–72·5%)	79·7% (77·8–81·4%)	75·4% (72·7–77·9%)	62·7% (59·5–65·7%)	35·3% (32·0–38·7%)	19·0% (16·1–22·2%)	59·9% (58·7–61·2%)	<0·0001
Anal sex in past year		17·0% (15·3–18·9%)	15·9% (14·4–17·5%)	12·7% (10·8–14·9%)	7·7% (6·1–9·5%)	3·6% (2·5–5·1%)	3·6% (2·4–5·3%)	10·5% (9·8–11·2%)	<0·0001
Genital contact without intercourse in past year		72·6% (70·4–74·8%)	77·1% (75·1–79·0%)	73·4% (70·6–76·0%)	62·4% (59·3–65·4%)	41·0% (37·6–44·4%)	28·1% (24·7–31·7%)	61·4% (60·2–62·6%)	<0·0001
**Masturbation**									
Masturbated in past 4 weeks		37·1% (34·7–39·5%)	43·7% (41·4–46·0%)	39·8% (36·8–42·8%)	37·6% (34·4–40·9%)	19·2% (16·7–22·0%)	10·3% (8·2–12·8%)	32·9% (31·7–34·1%)	<0·0001
**Sexual practices with female partners**									
Any sexual experience or contact with another woman		18·9% (17·1–20·9%)	18·1% (16·4–20·0%)	11·5% (9·8–13·6%)	8·8% (7·3–10·6%)	6·6% (5·1–8·4%)	2·6% (1·7–4·1%)	11·5% (10·7–12·3%)	<0·0001
Any sexual experience with genital contact with another woman		7·6% (6·4–9·0%)	8·8% (7·7–10·1%)	7·4% (6·0–9·2%)	6·6% (5·3–8·3%)	3·5% (2·4–5·0%)	0·8% (0·4–1·4%)	6·1% (5·6–6·7%)	<0·0001
At least one female sexual partner in past 5 years		6·2% (5·1–7·4%)	4·7% (3·9–5·6%)	3·5% (2·6–4·9%)	2·7% (1·9–3·8%)	1·0% (0·5–2·1%)	0·1% (0·0–0·5%)	3·2% (2·8–3·6%)	<0·0001
**Risk behaviours for HIV and sexually transmitted infections**‡									
Paid for sex§ in past 5 years		0%	0·1% (0·0–0·5%)	<0·1% (0·0–0·2%)	0·2% (0·0–1·2%)	0%	0·1% (0·0–1·0%)	0·1% (0·0–0·2%)	0·5366
At least one new sexual partner from outside the UK in past 5 years		8·6% (7·2–10·2%)	8·2% (7·0–9·6%)	3·2% (2·3–4·4%)	1·8% (1·1–2·7%)	1·2% (0·7–2·1%)	0·4% (0·1–1·5%)	4·1% (3·6–4·5%)	<0·0001
At least two sexual partners with whom no condom used in past year		14·3% (12·8–16·1%)	7·4% (6·3–8·7%)	3·6% (2·6–4·8%)	2·0% (1·4–3·0%)	0·8% (0·4–1·5%)	0·1% (0·0–0·6%)	4·9% (4·4–5·3%)	<0·0001
**Sexual attitudes**									
Non-exclusivity in marriage: always wrong		73·9% (71·9–75·8%)	71·2% (69·1–73·3%)	65·1% (62·1–68·0%)	60·6% (57·5–63·7%)	59·1% (55·8–62·3%)	58·2% (54·5–61·8%)	65·0% (63·8–66·2%)	<0·0001
One night stands: not wrong at all		8·8% (7·4–10·4%)	14·8% (13·2–16·5%)	14·9% (12·8–17·3%)	7·7% (6·3–9·4%)	4·0% (3·0–5·4%)	2·7% (1·7–4·2%)	9·3% (8·6–10·0%)	<0·0001
Male same-sex partnerships: not wrong at all		68·2% (65·9–70·3%)	66·8% (64·6–68·9%)	64·2% (61·0–67·3%)	60·4% (57·1–63·6%)	51·7% (48·3–55·0%)	40·3% (36·8–44·0%)	59·8% (58·6–61·1%)	<0·0001
Female same-sex partnerships: not wrong at all		67·3% (65·1–69·5%)	66·6% (64·4–68·7%)	64·5% (61·3–67·6%)	60·2% (56·9–63·4%)	52·0% (48·6–55·3%)	39·3% (35·8–42·9%)	59·6% (58·3–60·8%)	<0·0001
**Denominator**									
Unweighted		2140	2487	1215	1123	1030	874	8869	..
Weighted		1207	1380	1455	1443	1235	935	7654	..

Data are median (IQR), % (95% CI), or mean (SD), unless otherwise stated. All estimates are weighted. All participants (denominators vary across variables due to item non-response). Vaginal sex is defined as a man's penis in a woman's vagina.^21^ Oral sex is defined as mouth on a partner's genital area.^21^ Anal sex is defined as a man's penis in a partner's anus.^21^

* Not defined when asked in interview.

† Sexual intercourse defined as vaginal, oral, or anal sex; in participants who had at least one male sexual partner in the past year.

‡ With a female or male sexual partner, or both.

§ Vaginal, oral, or anal sex.

The reported number of lifetime sexual partners of the opposite sex by time of interview varied substantially by sex and age group (Table 2, Table 3). Overall, 94·5% of men and 95·8% of women aged 16–74 years reported at least one sexual partner of the opposite sex. The proportion reporting ten or more sexual partners was greater in men than in women, but the difference between the sexes was smaller as age decreased (Table 2, Table 3). Despite a shorter period of sexual activity, the proportion of women aged 16–24 years reporting ten or more male partners was more than twice that of those aged 65–74 years, and only slightly less than that of those aged 45–54 years (table 3).

82·1% of men and 77·7% of women reported at least one sexual partner of the opposite sex in the year before interview. The proportion peaked in men aged 35–44 years (92·5%) and women aged 25–34 years (91·8%). The proportions of men and women reporting at least one sexual partner of the opposite sex in the past year were largely similar in the three youngest age groups, but after the age of 55 years, men were more likely to have had a sexual partner of the opposite sex in the past year than were women (Table 2, Table 3). Participants aged 16–24 years were most likely to report two or more sexual partners of the opposite sex and at least one new sexual partner of the opposite sex in the past year (Table 2, Table 3). Nonetheless, at least 10% of men reported new sexual partners of the opposite sex in the past year until the age of 65 years (table 2), as did at least 11% of women until the age of 45 years (table 3).

We recorded substantial variability by age in the number of occasions of sexual intercourse with a partner of the opposite sex in the 4 weeks before interview in both men and women who reported at least one sexual partner of the opposite sex in the past year (Table 2, Table 3). The mean number of occasions (4·3 in men; 4·0 in women) was consistently higher than the median (three) across sexes and age groups (Table 2, Table 3). The lowest mean number was recorded for the oldest age groups in both sexes (Table 2, Table 3).

60·7% of participants reported having had vaginal sex in the past 4 weeks. This proportion decreased substantially after the age of 55 years in both sexes, as did the proportion reporting heterosexual oral sex in the past year (Table 2, Table 3). The proportion of participants reporting heterosexual anal sex in the past year decreased as age increased in both men and women (Table 2, Table 3). Overall, twice as many men than women reported masturbating in the past 4 weeks, with proportions decreasing steadily with age in men but falling only after the age of 55 years in women (Table 2, Table 3).

The proportion of participants of all ages reporting ever having a sexual experience or contact with an individual of the same sex was slightly higher in women (11·5%) than in men (8·0%; Table 2, Table 3). Additionally, the proportion hardly varied by age in men (table 2), but varied substantially in women, with the highest proportions reported for those aged younger than 35 years (table 3). These patterns were also recorded for proportions of men and women reporting ever having had a sexual experience with genital contact with an individual of the same sex or at least one sexual partner of the same sex in the past 5 years, although the proportions were lower than those reporting ever having had a sexual experience or contact with an individual of the same sex (Table 2, Table 3).

**Figure 1:**
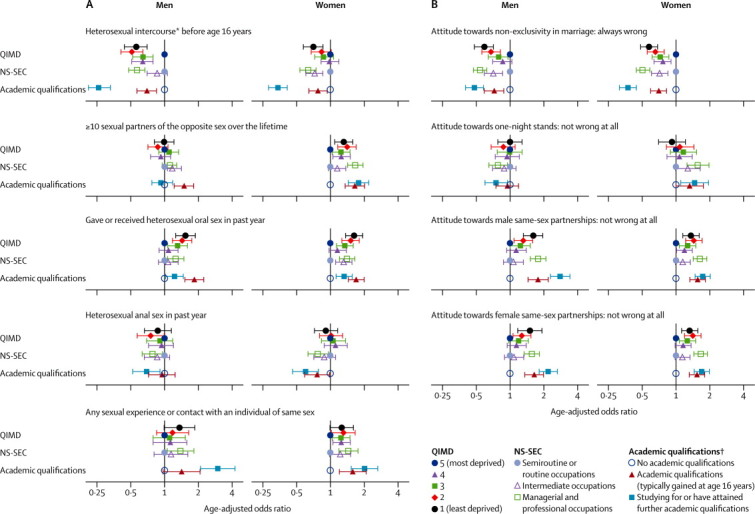
Association between key social determinants and (A) sexual behaviours and (B) attitudes in Natsal-3 participants aged 16–74 years, by sex Horizontal bars indicate 95% CIs. NS-SEC excludes people who are long-term unemployed and full-time students. Oral sex is defined as mouth on a partner's genital area.^21^ Anal sex is defined as a man's penis in a partner's anus.^21^ QIMD=Quintile of Index of Multiple Deprivation.^29^ NS-SEC=National Statistics Socio-Economic Classification.^28^ *Not defined when asked in interview. †Participants aged ≥17 years.

We generally recorded less variation in sexual behaviours and attitudes between groups of participants divided by area-level social-status measures than between those in groups divided by the two individual-level measures (figure 1). Of the individual-level measures, differences between groups were generally larger when divided by educational attainment than when divided by NS-SEC, although patterns were not consistent (figure 1). First heterosexual intercourse before age 16 years was significantly less frequent in participants with academic qualifications than in those with no academic qualifications, and less frequent in those in managerial and professional occupations than those in lower occupations (figure 1). However, the association was reversed in women for ten or more male sexual partners, after adjusting for age (figure 1). Any sexual experience or contact with an individual of the same sex was also associated with managerial and professional occupations and increased educational attainment (figure 1). We recorded a similar pattern in participants reporting heterosexual oral sex in the past year, but the pattern was reversed for heterosexual anal sex (figure 1). Although individuals with higher educational attainment and NS-SEC reported more tolerant attitudes towards non-exclusivity in marriage and same-sex partnerships than did those with lower academic attainment, the pattern was less clear in relation to attitudes towards one-night stands (ie, single sexual encounters; figure 1).

Odds of almost all sexual behaviours in participants aged 16–44 years were significantly lower in Natsal-1 than in Natsal-2 (table 4), which reflects the increase in risk behaviour between Natsal-1 and Natsal-2, as previously reported.^11^ The proportion reporting vaginal sex in the past 4 weeks was significantly lower in Natsal-3 than in Natsal-2 in both sexes, as was the number of occasions of heterosexual intercourse in the past 4 weeks (table 4), including in participants living with a partner (median five [IQR two to nine] to four [two to seven]). Additionally, in men, the proportion reporting at least one new female sexual partner in the past year was significantly lower in Natsal-3 (table 4).

**Table 4:** Change in numbers of sexual partners and prevalence of sexual practices, behaviours, and attitudes in Natsal-3 and Natsal-1 relative to Natsal-2, by sex

		**Men**			**Women**		
		Natsal-1	Natsal-2	Natsal-3	Natsal-1	Natsal-2	Natsal-3
**Sexual partners of the opposite sex**							
Number of partners over lifetime*							
	Mean (SD)	8·6 (33·1)	12·6 (35·5)	11·7 (21·6)	3·7 (13·2)	6·5 (9·7)	7·7 (16·2)
	Median (IQR)	4 (1–10)	6 (2–12)	6 (2–13)	2 (1–4)	4 (2–8)	4 (2–10)
	Age-adjusted odds ratio	0·68 (0·63–0·74)	1·00	0·92 (0·84–1·01)	0·44 (0·41–0·47)	1·00	1·18 (1·08–1·28)
Number of partners in past year*							
	Mean (SD)	1·2 (2·5)	1·5 (2·4)	1·5 (2·7)	1 (0·8)	1·2 (1·3)	1·3 (3·5)
	Median (IQR)	1 (1–1)	1 (1–1)	1 (1–1)	1 (1–1)	1 (1–1)	1 (1–1)
	Age-adjusted odds ratio	0·75 (0·69–0·83)	1·00	0·90 (0·81–1·00)	0·75 (0·68–0·81)	1·00	0·98 (0·89–1·09)
At least one new partner in past year		NA	29·9%	27·7%	NA	21·1%	22·2%
	Age-adjusted odds ratio	NA	1·00	0·85 (0·75–0·95)	NA	1·00	1·03 (0·91–1·15)
**Sexual practices with partners of the opposite sex**							
Number of occasions of sexual intercourse in past 4 weeks*†							
	Mean (SD)	6·4 (6·8)	6·2 (6·8)	4·9 (6)	6·1 (5·6)	6·3 (6·7)	4·8 (5·4)
	Median (IQR)	5 (2–9)	4 (2–9)	3 (1–7)	5 (2–8)	4 (2–9)	3 (1–7)
	Age-adjusted odds ratio	1·11 (1·01–1·21)	1·00	0·68 (0·62–0·75)	1·08 (1·00–1·16)	1·00	0·62 (0·57–0·67)
Vaginal sex in past month		72·1%	72·2%	68·0%	76·3%	76·1%	69·6%
	Age-adjusted odds ratio	1·06 (0·96–1·17)	1·00	0·84 (0·75–0·94)	1·05 (0·96–1·16)	1·00	0·72 (0·65–0·80)
Given or received oral sex in past year		69·7%	77·9%	77·1%	65·6%	76·8%	75·1%
	Age-adjusted odds ratio	0·66 (0·60–0·74)	1·00	0·97 (0·86–1·09)	0·58 (0·53–0·63)	1·00	0·91 (0·82–1·01)
Anal sex in past year		7·0%	12·2%	17·0%	6·5%	11·3%	15·1%
	Age-adjusted odds ratio	0·54 (0·46–0·62)	1·00	1·47 (1·28–1·68)	0·54 (0·47–0·62)	1·00	1·39 (1·23–1·57)
Genital contact without intercourse in past year		71·8%	78·4%	74·2%	68·0%	76·8%	75·3%
	Age-adjusted odds ratio	0·69 (0·62–0·77)	1·00	0·78 (0·69–0·89)	0·63 (0·57–0·68)	1·00	0·91 (0·82–1·01)
**Sexual practices with partners of the same sex**							
Any sexual experience or contact with partner of same sex		6·0%	8·4%	7·3%	3·7%	9·7%	16·0%
	Age-adjusted odds ratio	0·72 (0·61–0·85)	1·00	0·88 (0·73–1·07)	0·35 (0·30–0·42)	1·00	1·76 (1·54–2·01)
Any sexual experience with genital contact with partner of same sex		3·6%	5·4%	4·8%	1·8%	4·9%	7·9%
	Age-adjusted odds ratio	0·67 (0·54–0·82)	1·00	0·89 (0·71–1·12)	0·36 (0·29–0·45)	1·00	1·69 (1·43–2·00)
At least one sexual partner of the same sex in past 5 years		1·5%	2·5%	2·9%	0·8%	2·4%	4·7%
	Age-adjusted odds ratio	0·57 (0·42–0·78)	1·00	1·14 (0·85–1·53)	0·32 (0·22–0·46)	1·00	2·00 (1·59–2·51)
**Risk behaviours for HIV and sexually transmitted infections**‡							
Paid for sex§ in past 5 years		2·1%	4·3%	4·0%	NA	NA	0·1%
	Age-adjusted odds ratio	0·49 (0·38–0·62)	1·00	0·95 (0·75–1·20)	NA	NA	NA
At least one new sexual partner from outside the UK in past 5 years		NA	13·1%	11·7%	NA	6·9%	6·5%
	Age-adjusted odds ratio	NA	1·00	0·86 (0·73–1·00)	NA	1·00	0·92 (0·78–1·09)
At least two sexual partners with whom no condom was used in past year		NA	13·5%	10·7%	NA	7·5%	8·1%
	Age-adjusted odds ratio (95% CI)	NA	1·00	0·75 (0·64–0·87)	NA	1·00	1·06 (0·91–1·24)
Sexual attitudes*							
Non-exclusivity in marriage: always wrong		44·7%	51·2%	62·5%	53·2%	59·7%	69·8%
	Age-adjusted odds ratio	0·72 (0·66–0·79)	1·00	1·51 (1·36–1·67)	0·73 (0·67–0·78)	1·00	1·52 (1·39–1·67)
One night stands: not wrong at all		20·1%	27·3%	20·2%	5·4%	12·2%	13·0%
	Age-adjusted odds ratio	0·52 (0·48–0·57)	1·00	0·79 (0·72–0·86)	0·35 (0·33–0·38)	1·00	1·11 (1·03–1·20)
Male same-sex partnerships: not wrong at all		22·0%	36·1%	48·1%	28·1%	51·6%	66·3%
	Age-adjusted odds ratio	0·47 (0·43–0·52)	1·00	1·65 (1·50–1·82)	0·34 (0·32–0·37)	1·00	1·78 (1·63–1·94)
Female same-sex partnerships: not wrong at all		24·3%	41·2%	52·4%	27·7%	51·5%	66·1%
	Age-adjusted odds ratio	0·41 (0·37–0·44)	1·00	1·58 (1·44–1·75)	0·33 (0·31–0·36)	1·00	1·77 (1·62–1·93)

Data in parentheses are 95% CIs unless otherwise stated. All participants aged 16–44 years. Vaginal sex is defined as a man's penis in a woman's vagina.^21^ Oral sex is defined as mouth on a partner's genital area.^21^ Anal sex is defined as a man's penis in a partner's anus.^21^ NA=not available.

* Categorical levels modelled under the assumption of proportional odds.

† Sexual intercourse defined as vaginal, oral, or anal sex; in participants who had at least one sexual partner of the opposite sex in the past year.

‡ With a female or male sexual partner, or both.

§ Vaginal, oral, or anal sex.

In men, we noted little change between Natsal-2 and Natsal-3 in number of female sexual partners over the lifetime, number of female sexual partners in the past year, or numbers of individuals who had had a sexual experience with another man (table 4). The proportion of men reporting having had at least two sexual partners with whom condoms were not used in the past year decreased from Natsal-2 to Natsal-3 (table 4). Anal sex with a female partner in the past year was the only behaviour reported to increase in prevalence in men between the two surveys (table 4).

In women, we recorded increases between Natsal-2 and Natsal-3 in the reported number of male sexual partners over the lifetime, reporting of anal sex with a male partner in the past year, reporting of ever having had sexual experience or contact with another woman, reporting of ever having had a sexual experience with genital contact with another woman, and reporting of at least one female sexual partner in the past 5 years (table 4).

We noted increased acceptance of same-sex partnerships in men and women in Natsal-3 compared with Natsal-2, although women continued to be more accepting than men (table 4). By contrast, intolerance of non-exclusivity in marriage in both men and women was greater in Natsal-3 than in Natsal-2 (table 4). Tolerance of one-night stands decreased in men and increased in women between the two surveys (table 4).

**Figure 2:**
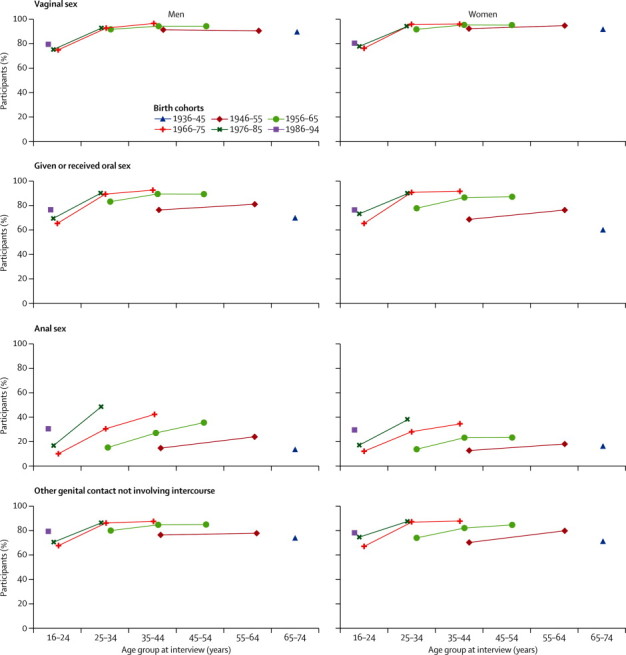
Participants from Natsal-1, Natsal-2, and Natsal-3 reporting heterosexual sexual practices at any point, by sex and 10-year birth cohort Each line connects values for the same birth cohort at different ages. Data for the 45–54 years age group missing for 1946–55 birth cohort, because only individuals aged 16–44 years were included in Natsal-2. Vaginal sex is defined as a man's penis in a woman's vagina.^21^ Oral sex is defined as mouth on a partner's genital area.^21^ Anal sex is defined as a man's penis in a woman's anus.^21^

**Figure 3:**
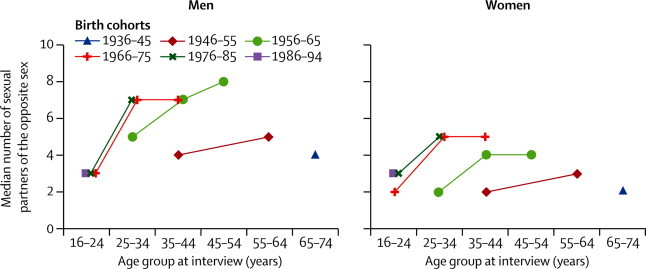
Number of sexual partners of the opposite sex over the lifetime reported by participants in Natsal-1, Natsal-2, and Natsal-3, by sex and 10-year birth cohort Each line connects values for the same birth cohort at different age groups. Data for the 45–54 years age group missing for 1946–55 birth cohort, because only individuals aged 16–44 years were included in Natsal-2.

Vaginal sex was almost universal in the group aged 25–34 years in all three birth cohorts for whom data about this age group were available (figure 2). By contrast, reporting of oral sex, other genital contact, and particularly anal sex with a partner of the opposite sex has increased in each successive birth cohort (figure 2). Therefore, as an example, less than 20% of both men and women born between 1946 and 1955 reported anal sex by the time they were aged 35–44 (ie, at the time of Natsal-1), but this proportion had increased to about 30% in those born a decade later (1956–65) and to almost 40% in those born 20 years later (1966–75) by the time they reached the same age (figure 2). In addition to increases in successive cohorts, increases between age groups within birth cohorts were also evident (figure 2). The median number of partners over the lifetime generally increased in successive birth cohorts in both men and women, but we recorded little cohort effect on partner numbers for the youngest age group at interview (16–24 years) representing those born between 1966 and 1994, especially in men (figure 3).

## Discussion

We have presented findings from Natsal-3 on sexual attitudes and lifestyles in Britain in 2010–12. By also including data from the two previous Natsal surveys^9^, ^10^, ^11^, ^12^ and thus responses from more than 45 000 people, we could track the sexual lifestyles of successive British birth cohorts back to the 1930s. We have shown that substantial changes have occurred in age at first heterosexual intercourse, numbers of sexual partners, sexual practices, and attitudes towards sex.

We have shown wide variability in sexual lifestyles by sex, age, and birth cohort, and, for the first time, have recorded behaviour patterns and attitudes in individuals aged up to 74 years. Most adults at all ages are sexually active, but sexual frequency and the range of practices reported reduces with age, especially in women. Although many aspects of health behaviour have strong social determinants,^31^ we recorded complex and inconsistent patterns, and noted that education is more strongly associated with sexual behaviours and attitudes than is individual socioeconomic status. Area-level deprivation was seldom associated with sexual behaviours.

Sexual frequency, and particularly reporting of recent vaginal sex, has decreased in the past decade. Increases in the reporting of oral sex with a partner of the opposite sex between Natsal-1 and Natsal-2 have not continued; prevalence was the same in Natsal-3 as in Natsal-2. By contrast, the reporting of anal sex with a partner of the opposite sex—albeit much lower than that of oral sex—increased in men and women between Natsal-1 and Natsal-2, and between Natsal-2 and Natsal-3. This finding suggests that heterosexual repertoires—or at least experimentation—have expanded in successive birth cohorts and over time.

More substantial changes in behaviour occurred between Natsal-1 and Natsal-2 than between Natsal-2 and Natsal-3, possibly because Natsal-1 coincided with public concern around the beginning of the HIV/AIDS epidemic. The start of the 1990s might have represented a low point in terms of sexual activity and risk behaviour, a hypothesis that is corroborated by the large decrease in diagnoses of sexually transmitted infections that occurred during the 1980s and early 1990s.^32^ Differences in patterns of sexual behaviour between men and women diminished between Natsal-1 and Natsal-2,^11^ but have decreased even further in Natsal-3 with respect to some sexual experiences and numbers of partners of the opposite sex. However, the proportion of women reporting sexual experience with same-sex partners now exceeds that of men, at least at younger ages, when the proportion describing themselves as bisexual is highest.

As has been reported elsewhere,^33^ and congruent with continuing liberalising legislative changes in sexual orientation and expression of sexuality,^34^, ^35^, ^36^, ^37^ we recorded increased acceptance of same-sex partnerships in men and women in successive Natsal surveys. However, there is now greater disapproval of non-exclusivity in marriage and of one-night stands by men. Although women continue to be more disapproving than men are of both these behaviours, women continue to be more accepting of same-sex partnerships.

Although response in the previous Natsal surveys was higher than for Natsal-3 (66·8% for Natsal-1^9^, ^10^ and 65·4% for Natsal-2^11^, ^23^), response rates for social surveys in Britain have decreased in the past decade,^38^ and different sampling strategies and changing industry standards for calculation of response make direct comparisons with other surveys, including Natsal-1 and Natsal-2, difficult. However, the response in Natsal-3 is in line with other major social surveys completed in Britain around the same time.^33^, ^39^ Nonetheless, we acknowledge that non-response could be a source of bias for our data. We aimed to minimise this bias by weighting the sample so that it was broadly representative of the underlying population with respect to the distribution of the sexes, age, and regions as used in the census. Furthermore, the sampling strategy used for the Natsal studies means that the target population is specifically the population resident in private households in Britain, and as such excludes individuals living in institutions, whose behaviour could differ from others, such that this strategy is also a potential source of bias.

Caution is needed when interpreting changes in behaviour captured by cross-sectional surveys like Natsal. Behaviour change and differences between men and women should be considered in the context of changing social attitudes and norms, which can affect willingness to report and social desirability bias. The hypothesis that changing attitudes and norms affects willingness to report and social desirability bias has been examined elsewhere.^40^, ^41^ By contrast with comparisons between Natsal-1 and Natsal-2, which suggested that willingness to report might have increased in Natsal-2 because of improvements in methods,^24^, ^40^ we noted little evidence of such a difference in a similar comparison between Natsal-2 and Natsal-3.^41^ We partly attribute this finding to fewer methodological differences between the latest two surveys than between Natsal-1 and Natsal-2, because we used computer-assisted personal interview and self-interview for both Natsal-2 and Natsal-3 (which have contributed to low levels of item-non-response; typically 1–3%^21^), together with consistent question wording across all three surveys.

Although national surveys of sexual behaviour have been done in many other countries (panel),^42^, ^43^, ^44^, ^45^, ^46^ differences in methods, specifically the absence of serial surveys with similar methods in the same population, restrict the extent to which international comparisons are possible, especially for investigation of changes over time. Although behaviour in the USA seems not to have changed in the past decade,^45^ increased experimentation with sexual repertoires and sexual orientation, especially by women, and a narrowing of the gap between the sexes recorded for Britain in Natsal is consistent with findings from the French national probability surveys of sexual behaviour.^46^ Therefore, the Natsal data are useful and their implications relevant internationally as well as nationally.

Panel **Research in context**

**Systematic review**

National surveys of sexual behaviour of varying size and quality have been completed in an increasing number of countries.^42^ However, Britain (ie, England, Scotland, and Wales) is one of a very small number of countries where three cross-sectional probability sample surveys of sexual attitudes and lifestyles with similar methods have been completed, as far as we are aware.

**Interpretation**

In this Article, we have presented findings from the three National Surveys of Sexual Attitudes and Lifestyles in Britain. Here, we have shown that sexual lifestyles have changed substantially in the past 60 years. Age at first sexual intercourse in people in Britain has decreased, increasing the time available to accumulate sexual partners, as evident from increases in the number of partners reported in recent decades, especially in women. Sexual activity continues into later life, albeit diminished in range and frequency, emphasising that attention to sexual health and wellbeing is needed throughout the life course. We acknowledge that changes in attitudes towards sex and sexual partnerships could affect willingness to report sensitive behaviours, but our analyses suggest that this effect is likely to be negligible. Therefore, we conclude that the reported changes in Natsal-3 reflect real changes in the behaviour of the British population.

The recorded trends need to be considered against the backdrop of changing social norms, demographic trends, and changing legislation and policy. In Britain, as in many other countries, the position of women in society—particularly their increased social, economic, and reproductive freedom—has continued to change. The proportion of women who were married or cohabiting decreased substantially between the three surveys, and the intervals between first heterosexual intercourse, first cohabitation, and birth of first child have grown.^47^ The portrayal of women's increasing independence and choice of diverse sexual lifestyles in the media could have increased both inclination to engage in, and willingness to report, experiences. The demographic and social changes provide new opportunities for women and their sexual lifestyles, as shown by the increased numbers of partners and greater likelihood of same-sex experience reported by Natsal-3 participants.

The decrease in sexual frequency and recent vaginal sex since Natsal-2 also needs to be set in demographic context. The proportion of people not living with a partner has increased since 1996, because of an increase in the proportion who marry late in life or not at all, or who experience breakdown of relationships.^48^ However, because sexual frequency in individuals living with a partner also dropped during this time, people in Britain seem to report sex less frequently nowadays, even taking account of changes in the nature of sexual partnerships.

Despite an increase in the proportion of the population not living with a partner and thus potentially seeking to form new partnerships, as well as the new opportunities for people to meet and interact (eg, via social media and the internet), we recorded little change in partner acquisition since the increase between Natsal-1 and Natsal-2.^11^ Reporting of new partners remains highest in individuals younger than 25 years, which is consistent with prevalence estimates^49^ and surveillance^4^ of sexually transmitted infections. Nonetheless, partnership formation continues throughout the life course, especially for men, so sexual health advice should not exclusively target young people. Indeed, the finding that a higher proportion of men older than 50 years than women of this age report sex in the past year largely reflects how these women are more likely to be living without a partner because of widowhood or divorce and subsequently having no new partner.^26^, ^27^, ^48^

The large changes in sexual lifestyles that have occurred in the past 60 years that have been captured by the three Natsal studies^9^, ^10^, ^11^, ^12^, ^13^ present both public health challenges and opportunities. Data from our latest survey suggest that levels of sexual activity and diversity have been sustained but have not increased, at least in men. They signal the need for sex education, health policy, and practice that recognises and responds to increased sexual diversity in the population. Our findings have a positive message for sexual health interventions because, if behaviour is malleable, then the potential to modify it to improve sexual health status could be increased. Public health programmes need to embrace the evidence of change to ensure that services and preventive interventions are appropriate for present lifestyles; that they promote informed, consensual, safe, respectful, and pleasurable relationships; and that their aims are consistent with a broader definition of sexual wellbeing.^50^

Examination of the sexual lifestyles of older as well as younger people in Britain emphasises the importance of sexual health and wellbeing across the life course, and underscores the limitations of restricting sexual health policy and services to younger age groups. Although the sexual health and wellbeing needs of the British population might have diversified, they remain as important as when the national strategies for sexual health were launched earlier in the new millennium.^15^, ^16^, ^17^

## Supplementary Material

Supplementary appendix

Supplementary video Results of over 45 000 respondents, recording the dramatic changes in our sexual lifestyles over the past 60 years.
