# Supplementary material for: Changes in sexual attitudes and lifestyles in Britain through the life course and over time: findings from the National Surveys of Sexual Attitudes and Lifestyles (Natsal)
Source: Lancet. 2013 Nov 30;382(9907):1781–94. doi: 10.1016/S0140-6736(13)62035-8 (PMC3899021; doi:10.1016/S0140-6736(13)62035-8)
Supplement: Supplementary appendix [file mmc1.pdf]

THE LANCET

Supplementary appendix

This appendix formed part of the original submission and has been peer reviewed. We post it as supplied by the authors.

Supplement to: Mercer CH, Tanton C, Prah P, et al. Changes in sexual attitudes and lifestyles in Britain through the life course and over time: findings from the National Surveys of Sexual Attitudes and Lifestyles (Natsal). *Lancet* 2013; published online Nov 26. http://dx.doi.org/10.1016/S0140-6736(13)62035-8.

Changes in sexual attitudes and lifestyles in Britain through the life course and over time: findings from the National Surveys of Sexual Attitudes and Lifestyles (Natsal)

Web appendix: Comparison of Natsal-3 weighted sample with 2011 Census population figures

	Natsal-3 sample after selection weighting (%)			Natsal-3 sample after final weighting (%)			2011 Census figures (%)		
**Age**	**Men**	**Women**	**All**	**Men**	**Women**	**All**	**Men**	**Women**	**All**
16-19	7.8	6.6	7.1	7.1	6.7	6.9	7.1	6.7	6.9
20-24	8.9	8.0	8.4	9.4	9.1	9.3	9.4	9.1	9.3
25-29	8.3	8.5	8.4	9.3	9.2	9.3	9.3	9.2	9.3
30-34	7.9	8.4	8.2	9.0	8.8	8.9	9.0	8.8	8.9
35-39	8.5	9.8	9.3	9.0	9.0	9.0	9.1	9.0	9.0
40-44	9.6	10.7	10.2	9.9	10.0	10.0	9.9	10.0	10.0
45-54	18.7	20.4	19.7	18.8	18.9	18.8	18.8	18.9	18.8
55-64	16.8	16.2	16.5	15.9	16.1	16.0	15.9	16.1	16.0
65-74	13.5	11.4	12.3	11.5	12.2	11.8	11.5	12.2	11.8
**Row %**	**42.6**	**57.4**		**49.5**	**50.5**		**49.5**	**50.5**	
**Government Office Region**	**Men**	**Women**	**All**	**Men**	**Women**	**All**	**Men**	**Women**	**All**
North East	5.1	4.9	5.0	4.2	4.3	4.3	4.2	4.3	4.3
North West	12.9	13.1	13.0	11.5	11.5	11.5	11.5	11.5	11.5
Yorkshire & the Humber	8.2	8.0	8.1	8.6	8.6	8.6	8.6	8.6	8.6
East Midlands	8.6	7.9	8.2	7.4	7.4	7.4	7.4	7.4	7.4
West Midlands	8.6	9.1	8.9	9.1	9.0	9.0	9.1	9.0	9.0
South West	8.6	8.2	8.4	8.6	8.5	8.6	8.6	8.5	8.6
East	10.7	10.4	10.5	9.4	9.4	9.4	9.4	9.4	9.4
Inner London	3.6	3.7	3.7	4.9	5.5	5.2	5.6	5.5	5.6
Outer London	7.1	7.0	7.1	8.6	8.1	8.3	8.0	8.1	8.0
South East	13.1	14.2	13.7	13.9	13.9	13.9	13.9	13.9	13.9
Wales	5.5	5.1	5.3	5.0	5.0	5.0	5.0	5.0	5.0
Scotland	7.9	8.4	8.1	8.7	8.9	8.8	8.7	8.9	8.8
